# Associations between upper quarter Y-balance test performance and sport-related injuries in adolescent handball players

**DOI:** 10.3389/fspor.2023.1076373

**Published:** 2023-04-03

**Authors:** Julian Bauer, Stefan Panzer, Markus Gruber, Thomas Muehlbauer

**Affiliations:** ^1^ Division of Movement and Training Sciences, Biomechanics of Sport, University of Duisburg-Essen, Essen, Germany; ^2^ Department of Sport Science, Human Performance Research Centre, University of Konstanz, Konstanz, Germany; ^3^ Institute of Sport Science, Saarland University, Saarbrücken, Germany

**Keywords:** epidemiology, upper extremity, shoulder mobility/stability, youth, asymmetry

## Abstract

Handball players are at a high risk of suffering a sport-related injury. Recent studies in various adult populations (e.g., US Army soldiers/warrior athletes, and military members) showed that poor scores in the upper quarter Y-balance test (YBT-UQ) are related to an increased risk of injury. Yet, it is unclear whether this also applies to adolescent handball players. Thus, the present study aims to determine if pre-season YBT-UQ performance is associated with sport-related injuries during the competitive season in adolescent handball players. One hundred and thirty-three adolescent handball players (age: 15.4 ± 1.7 years; m = 99, f = 42) who competed in the second highest league in the Rhine-Ruhr region, Germany, during the 2021/2022 season participated in the study. Before the competitive season, the players performed the YBT-UQ to assess upper extremity mobility and stability of the throwing and non-throwing arm. Over the 8-month competitive season, the coaches monitored the occurrence of sports-related injuries once a week, using an injury report form from the legal accident insurance. Fifty-seven players (43%) incurred a sport-related injury during the competitive season, of which 27 (47%) had upper body injuries, and 30 (53%) were lower body injuries. The YBT-UQ performance of the throwing and non-throwing arm did not significantly differ between injured and non-injured players. Further, Cox proportional hazard survival regression model analyses revealed that only the presence of an inferolateral reach asymmetry score ≥7.75% arm length was associated with a moderate increase in the risk (hazard ratio = 2.18, 95% confidence interval = 1.02–4.68, *p* = 0.045) of lower but not upper or whole-body injuries. Our findings suggest that the YBT-UQ has limited value as a field-based screening tool to assess the risk of sport-related injuries in adolescent handball players.

## 1. Introduction

Handball is an Olympic team sport played worldwide and is especially popular in Europe (1). It is played in 199 countries and has approximately 19 million registered players (2). It is a team sport with high physical demands, and body contact actions are an integral part of the game (3, 4). These body contacts mainly happen during throwing attempts by offensive players and tackles by defending players (5). To interrupt the flow of play, body actions against the opponent are highly important (6, 7). Thus, it comes as no surprise that the International Olympic Committee ranks handball as one of the Olympic sports with the highest injury rates (8, 9), with adolescent handball players ranging from 8.3 injuries/1,000 match hours in males to 14.5 injuries/1,000 match hours in female U17 players (3). Møller et al. (4) reported an injury incidence of 11.5 in male and 10.8 in female U16 players, as well as 13.0 in female and 17.2 in male U18 players per 1,000 match hours. Asai et al. (10) even reported a higher number of match injuries in 13- to 14-year-old players, with 26.5 injuries/1,000 match hours, further differentiating between boys (32.7 injuries/1,000 match hours) and girls (20.1 injuries / 1,000 match hours). However, it must be kept in mind that different injury definitions exist (6, 9), which makes comparisons between studies difficult.

Injuries have adverse effects, as injured players cannot develop individually and cannot contribute to their team's success (11). In addition, the subsequent costs of injuries are a high economic burden (12), with professional handball players reporting mean costs of 1,100 € per injury, and injuries in amateur players often resulting in a temporary inability to work (13). Therefore, preventing injuries in youth handball is a key priority for practitioners and scientists alike (11, 14).

In conjunction with the high injury rates due to the game’s physical nature, handball is also characterized by unilateral movements. Throwing and passing techniques are predominantly executed with one side of the body, i.e., the throwing arm (15). This functional specialization may lead to distinct adaptations, such as differences between the throwing and non-throwing arm in terms of stability, mobility, or throwing velocity (15), but also motor control, performance outcome, and skill acquisition (16). These diverging adaptations may result from the asymmetrical distribution of load and the associated neuromuscular stimuli. In this context, exceeding a specific cut-off value between the throwing and non-throwing arm in terms of reach differences, i.e., upper extremity asymmetry, may pose a potential risk factor for injuries (17, 18). In addition, pronounced asymmetries have also been reported as having detrimental effects on physical performance (19). Preseason assessments may therefore be reasonable to identify injury risk factors (14). One suitable and highly reliable test that has the potential to assess upper extremity mobility and stability, as well as asymmetries, is the upper quarter Y-balance test (YBT-UQ) (20, 21). Different authors have already examined whether preseason YBT-UQ performance is related to injuries during the competitive season. Teyhen et al. (18) investigated the risk of suffering a time-loss injury in 922 US Army soldiers/warrior athletes (age: 24.7 ± 5.2 years). A maximum superolateral (SL) reach distance of ≤80.1% arm length (AL) as well as an inferolateral (IL) reach asymmetry of ≥7.75% AL was found to increase the likelihood of a future time-loss injury. Further, specific cut-off values for military personnel (*N *= 494, m = 454, f = 40; age: 28.6 ± 6.8 years) were reported by Campbell et al. (22). In this study, the authors identified an SL reach distance of ≤57.75 cm and a composite score (CS) of less than ≤81.1% AL in the YBT-UQ as risk factors for suffering an upper quadrant musculoskeletal injury.

In addition, Cosio-Lima et al. (23) assessed the association between YBT-UQ performance and physical training-related injuries in male Coast Guard Maritime Security Response Team candidates (*N *= 31, age: 24 ± 4 years). The odds ratio (OR) to suffer an injury was 5.4 in the case of a CS of 81.8–89.3% AL, and an OR = 3.6 in the case of a CS of 89.0–99.0% AL for the left arm. For the right arm, an OR = 5.4 was present in the case of a CS 77–88.9% AL, and an OR = 3.6 in the case of a CS of 89.0–102.7% AL. The authors concluded that lower scores in the YBT-UQ were associated with an increased injury risk. In the most similar group to handball players, Bennett et al. (24) assessed preseason YBT-UQ performance in 257 elite adolescent male Australian Football players (age: 17.1 ± 0.8 years) to determine the relationship with in-season injuries. The authors did not detect significant associations between prospective upper quarter body injury risk and the YBT-UQ values.

Drawing upon these findings, the YBT-UQ, especially the SL reach direction and a high IL reach asymmetry, may be associated with future time-loss injuries in general. Because studies assessing the injury risk in adolescent handball players based on upper extremity mobility and stability performance are lacking, the present study aims to investigate whether preseason YBT-UQ performance is associated with the occurrence of sport-related injuries during the competitive season in this group. Our original contribution to the field was to determine whether a relationship exist between YBT-UQ values and handball-specific injuries. In this context, it will be assessed whether surpassing the cut-off values in terms of asymmetry or YBT-UQ scores that were reported in other cohorts (18, 22) also poses a risk [increased hazard ratio (HR)] in adolescent handball players. Confirming predefined YBT-UQ cut-off values that increase the likelihood of injuries may help practitioners develop intervention programs that specifically target these weaknesses in terms of total YBT-UQ scores and asymmetries. We hypothesized that injured players would show significantly worse results in the YBT-UQ and a significantly increased HR when (a) the SL reach distance was ≤80.1% AL, (b) the SL reach distance was ≤57.75 cm, (c) the CS was ≤81.1% AL, or (d) the IL reach asymmetry exceeded ≥7.75% AL.

## 2. Methods

### 2.1. Participants

The sample consisted of previously (at least 2 weeks before the baseline assessment) non-injured sub-elite adolescent handball players (Table 1). Before the study, different female and male teams of the Rhine-Ruhr region, Germany, were contacted, and their coaches were asked if they were interested in participating in the study. Similar training regimens (i.e., training frequencies of 3–4 times per week), playing levels (i.e., regional), and age ranges (i.e., adolescence) across the participating teams were ensured by the examiner of the study. Parents or legal guardians were informed about the goals of the study, possible risks, and the testing procedures. In addition, the written and informed consent of all participants and their parents or legal guardians was obtained before any assessment. The exclusion criteria were vestibular, visual, or proprioceptive disorders; and functional limitations that could affect YBT-UQ performance (15). All coaches and participants were informed about the possibility of discontinuing participation in the study at any given time. The study was carried out according to the Declaration of Helsinki (24). It was approved by the Ethics Committee of the Faculty of Social Sciences at the University of Duisburg-Essen (TM_23.03.2020).

**Table 1:** Characteristics of the study participants (*N *= 133).

Variable	Non-injured (*n *= 76)	Injured (*n *= 57)	*p*-Value
Age (years)	15.7 ± 1.7	15.7 ± 1.7	0.070
Sex (f/m)	22/54	20/37	—
Body height (m)	1.75 ± 0.09	1.72 ± 0.09	0.102
Body mass (kg)	70.2 ± 16.5	64.9 ± 12.8	0.046
BMI (kg/m^2^)	22.8 ± 4.2	21.5 ± 3.3	0.100
Arm dominance (l/r)	11/65	9/48	—
Throwing arm (l/r)	9/67	8/49	—
Throwing arm length (cm)	89.2 ± 5.6	87.4 ± 5.9	0.067
Non-throwing arm length (cm)	88.9 ± 5.5	87.1 ± 5.9	0.073
Training experience (years)	8.1 ± 3.1	8.6 ± 2.6	0.405

Data are group mean values ± standard deviations. BMI, body mass index; f, female; l, left; m, male; r, right.

### 2.2. Testing procedures

#### 2.2.1. Assessment of anthropometric variables

Body height, mass, and upper-limb length were assessed before the testing. The upper-limb length was measured from the distal tip of the middle finger, with the shoulder at 90-degree abduction (26), to the seventh cervical spinous process (C7). Body height was measured using a Seca 217 (Seca, Basel, Switzerland) linear measurement scale (to the nearest 0.1 cm), with participants standing straight and upright without shoes. Body mass was measured with a Seca 803 (Seca, Basel, Switzerland) electronic scale (to the nearest 100 g) wearing light sportswear and without shoes. The body mass index (BMI) was calculated by dividing body mass by the squared of body height (kg/m^2^). Training experience in years as well as the dominant arm and the throwing arm, were requested from all participants and recorded by the examiner.

#### 2.2.2. Assessment of shoulder mobility and stability

The YBT-UQ was executed with a Y Balance Test Kit (Move2Perform, Evansville, IN, United States). A specific YBT-UQ testing protocol was used to assess the maximal reach in the different reach directions of each participant. The examiners were either graduates of sports sciences or physiotherapists and were accustomed to executing the YBT-UQ. The correct execution of the tests was demonstrated by one of the examiners before the actual tests were carried out. All subjects had to start in a push-up position (26) with the 3rd metacarpophalangeal joint at the centre of the device (27) and their feet shoulder width apart. The right arm was always the first stance arm, and the mobile indicator had to be moved by the left arm in the medial (MD), IL, and SL reach directions consecutively, with no breaks in between movements. After three trials with the right arm as the stance arm, the same procedure was repeated, with the left arm as the stance arm and the right arm as the mobile arm. The three-point contact (left and right foot on the floor, stance arm on the testing device) also had to be held throughout each trial. Trials were valid if the subjects consistently maintained the push-up position with the stance arm on the testing device and did not actively push the indicator (i.e., held contact with the indicator until the final position). Subjects had a 30 s rest period in between trials of the right and left arm. Only the best scores from each reach direction were taken for further analyses (26). A CS was calculated as the mean of the averaged maximal distances of the best trials for all three reach directions, normalized to AL.

#### 2.2.3. Injury surveillance

All players and their respective coaches agreed to continuous monitoring of injuries. As the YBT-UQ assesses mobility and stability of the whole kinetic chain (28), and deficits in any of the kinetic chain segments can cause a break that may increase the likelihood of injuries (29), we decided to assess all injuries together (Table 2) and in addition, upper (Table 3) and lower (Table 4) body injuries separately. Within-sub-analyses, the location of the injuries was differentiated between upper (above the waist—arms, shoulders, torso, and head) and lower (below the waist—legs, feet, and hip) body injuries (30) segments can a break that may increase the likelihood of injuries. Injury reports were submitted weekly and accurately tracked throughout the season. Coaches transferred any injuries to the assessor of this study via email or phone using an injury report form from the legal accident insurance. The season lasted approximately 8 months, from October to April/May, with weekly matches only interrupted by the public school holidays. This led to between 16 and 26 matches for each team throughout the season, depending on the number of teams in their respective leagues. All coaches were informed about the definition of upper or lower body injury location before the study to include and differentiate these within the injury registration sheet. All data were pseudonymized and stored in a database. A time-loss injury definition (31) was chosen based on the proposal of the International Olympic Committee (32). Specifically, a time-loss injury is defined as a physical complaint that results in an athlete missing a training session or match (33). Players were categorized as “injured” if they suffered at least one time-loss injury over the course of the season. All other players were classified as “non-injured,” and the subsequent analyses were performed based on this dichotomous categorization (Tables 1 and 5).

**Table 5:** Normalized upper quarter Y-balance test reach distances and asymmetry scores of adolescent handball players.

Variable	Non-injured (*n *= 76)	Injured (*n *= 57)	*p*-Value
Throwing arm reach			
Medial reach (% AL)	110.2 ± 11.3	109.2 ± 10.5	0.293
Inferolateral reach (% AL)	105.6 ± 14.0	104.7 ± 11.7	0.361
Superolateral reach (% AL)	85.8 ± 11.7	86.6 ± 11.2	0.348
Composite score (% AL)	100.5 ± 10.0	100.2 ± 8.9	0.415
Non-throwing arm reach			
Medial reach (% AL)	108.1 ± 9.8	107.3 ± 8.7	0.313
Inferolateral reach (% AL)	104.9 ± 14.5	107.2 ± 13.1	0.179
Superolateral reach (% AL)	83.6 ± 12.8	84.4 ± 11.5	0.356
Composite score (% AL)	98.9 ± 9.8	99.7 ± 8.6	0.323
Asymmetry scores			
Medial reach (% AL)	6.0 ± 4.9	5.9 ± 4.6	0.898
Inferolateral reach (% AL)	8.1 ± 7.6	8.3 ± 6.3	0.854
Superolateral reach (% AL)	6.1 ± 5.4	7.0 ± 5.1	0.312
Composite score (% AL)	4.2 ± 3.9	4.4 ± 3.6	0.751

Data are group mean values ± standard deviations. AL, arm length.

**Table 6:** Location^a^ and frequency of sport-related injuries observed during the competitive season.

Injury location	Injury frequency, *n* (%)
Upper body (i.e., head, torso, shoulder, and arm)	27 (47)
Lower body (i.e., hip, leg, and foot)	30 (53)

^a^ The assignment of injury location was performed in accordance with Gardner et al. (30).

**Table 2:** Results of the Cox proportional hazard survival regression for sport-related injuries of the whole body by throwing arm.

Variable	*B*	HR	95% CI	*p*-Value
Throwing arm reach				
Superolateral reach distance ≤80.1% AL^a^	−0.208	0.81	0.45–1.47	0.491
Superolateral reach distance ≤57.75 cm^b^	—	—	—	—
Composite score ≤81.1% AL^b^	—	—	—	—
Non-throwing arm reach				
Superolateral reach distance ≤80.1% AL^a^	0.420	1.52	0.84–2.75	0.162
Superolateral reach distance ≤57.75 cm^b^	0.985	2.68	0.62–11.54	0.187
Composite score ≤81.1% AL^b^	—	—	—	—
Asymmetry				
Inferolateral reach asymmetry ≥7.75% AL^a^	0.102	1.11	0.66–1.87	0.703

AL, arm length; CI, confidence interval; HR, hazard ratio.

^a^ Reported by Teyhen et al. (18).

^b^ Reported by Campbell et al. (22).

**Table 3:** Results of the Cox proportional hazard survival regression for sport-related injuries of the upper body (i.e., arm, shoulder, torso, and head) by throwing arm.

Variable	*B*	HR	95% CI	*p*-Value
Throwing arm reach				
Superolateral reach distance ≤80.1% AL^a^	−0.202	0.82	0.35–1.89	0.636
Superolateral reach distance ≤57.75 cm^b^	—	—	—	—
Composite score ≤81.1% AL^b^	—	—	—	—
Non-throwing arm reach				
Superolateral reach distance ≤80.1% AL^a^	0.556	1.74	0.77–3.97	0.185
Superolateral reach distance ≤57.75 cm^b^	0.859	2.36	0.51–10.95	0.273
Composite score ≤81.1% AL^b^	—	—	—	—
Asymmetry				
Inferolateral reach asymmetry ≥7.75% AL^a^	−0.642	0.53	0.23–1.23	0.138

AL, arm length; CI, confidence interval; HR, hazard ratio.

^a^ Reported by Teyhen et al. (18).

^b^ Reported by Campbell et al. (22).

To ensure high compliance, coaches who missed submitting the information were actively contacted by the assessor to ensure consistent monitoring of the injury data. To gather all information in a timely manner, an alternative contact person from the coaching staff of each team was identified and contacted in case of delays.

### 2.3. Statistical analyses

Descriptive statistics were calculated for group mean values and standard deviations using Statistical Package for Social Sciences version 28.0 (IBM Corp., Armonk, NY, United States), which was used for all analyses. The sample size was estimated based on an *a priori* power analysis with G*Power (34). The analysis was run with *ω* = 0.3, *α* = 0.05, 1-*β* = 0.80, *df* = 2 which resulted in a total sample size of *N* = 108 participants. Cox proportional hazard survival regression models were applied for the whole, upper, and lower body to investigate the relationship between the dependent variable (i.e., time to first injury) and the following *a priori* predictor variables: IL reach asymmetry (two levels: ≥7.75% AL and <7.75% AL); normalized SL reach distance (two levels: ≤80.1% AL and >80.1% AL); absolute SL reach distance (two levels: ≤57.75 and >57.75 cm); normalized CS (two levels: ≤81.1% AL and >81.1% AL). In accordance with Hopkins (35), effect sizes were quantified using HR and considered trivial (≤1.29), small (1.30–1.99), moderate (2.00–3.99), or large (≥4.00). For all analyses, a *p*-value of <0.05 was considered statistically significant.

## 3. Results

Of the 133 players assessed, all players were tracked over the course of the entire season. There were no dropouts, and only one player changed clubs during the season. However, data on this player were still accessible, as he changed from one club participating in the study to another. Overall, the coaches’ response rate throughout the season was 100%, indicating that all data and weekly reports were provided to the examiner. No significant differences in preseason YBT-UQ performance were found between injured and non-injured players for the throwing and the non-throwing arm reach (Table 5).

Fifty-seven players (43%) incurred a sport-related injury during the competitive season, of which 27 (47%) were upper body injuries and 30 (53%) lower body injuries (Table 6). In terms of asymmetry scores (Table 5), no significant differences were present in either reach score (MD: *p* = 0.898; IL: *p* = 0.854; SL: *p* = 0.312; CS: *p* = 0.751). In total, 14 out of the 57 injuries occurred during matches, while the remaining 43 occurred during training sessions. Based on the total match hours of all assessed players (3,134 match hours), this corresponds to a match injury rate of 4.5 injuries/1,000 match hours.

**Table 4:** Results of the Cox proportional hazard survival regression for sport-related injuries of the lower body (i.e., hip, leg, and foot) by throwing arm.

Variable	*B*	HR	95% CI	*p*-Value
Throwing arm reach				
Superolateral reach distance ≤80.1% AL^a^	−0.227	0.80	1.02–4.68	0.607
Superolateral reach distance ≤57.75 cm^b^	—	—	—	—
Composite score ≤81.1% AL^b^	—	—	—	—
Non-throwing arm reach				
Superolateral reach distance ≤80.1 AL%^a^	0.257	1.29	0.54–3.10	0.565
Superolateral reach distance ≤57.75 cm^b^	—	—	—	—
Composite score ≤81.1% AL^b^	—	—	—	—
Asymmetry				
Inferolateral reach asymmetry ≥7.75% AL^a^	0.779	2.18	1.02–4.68	0.045

AL, arm length; CI, confidence interval; HR, hazard ratio.

^a^ Reported by Teyhen et al. (18).

^b^ Reported by Campbell et al. (22).

The results of the Cox proportional hazard survival regression models are shown in Tables 2–4. The analyses revealed that only the presence of an IL with an asymmetry score ≥7.75% AL was associated with a moderate increase in the risk [HR = 2.18, 95% confidence interval (CI) = 1.02–4.68, *p* = 0.045] of lower body injuries (i.e., hip, leg, and foot) (Table 4).

## 4. Discussion

The study aims to investigate if preseason YBT-UQ performance is associated with sport-related injuries during the competitive season in adolescent handball players. The main results can be summarized as follows: (a) no significant differences in preseason YBT-UQ scores were found between injured and non-injured players both for the throwing as well as the non-throwing arm; (b) YBT-UQ distance and asymmetry cut-off values (except IL reach asymmetry) could not be confirmed as factors for an increased risk of sport-related injuries in the present target group.

Our results align with those of Bennett et al. (24), who also did not find significant associations between upper quarter body injury risk and the modified YBT-UQ values. However, our findings contradict those of Cosio-Lima et al. (23), Teyhen et al. (18), and Campbell et al. (22), who reported an increased risk of injuries when specific YBT-UQ cut-off values were surpassed. In addition, we did not find significant differences in our sub-analyses which differentiated between upper and lower body injuries. In contrast to Campbell et al. (22), we did not find a significant relationship between the YBT-UQ cut-off values and injury incidence in the upper body. However, Campbell et al. (22) assessed adult (28.6 ± 6.8 years) military personnel who, contrary to adolescent handball players, might be more susceptible to the reported cut-off values, as the duration of the exposure to potential risk factors in terms of years and overall load is much higher in a military cohort compared with an adolescent group of handball players.

Several reasons may explain the lack of relationships between YBT-UQ performance and sport-related injuries. The study by Campbell et al. (22) used a different assessment technique than the present study, as in their study only upper quadrant musculoskeletal injuries were assessed. Therefore, the proposed risk factors of age, sex, and cohort may be subject-related moderator variables. However, based on our sub-analysis on upper body injuries, we could not confirm the results of Campbell et al. (22), as our results did not show any associations between upper body injuries and YBT-UQ results. Further, adolescent handball players have had lower exposure to the specific demands of handball training (i.e., overall training load) compared with senior players (36). Therefore, they may not be as susceptible to the proposed risk factors as an adult military/combat cohort. Contrary to our expectations, however, the Cox proportional hazard survival regression model revealed a relationship between IL reach asymmetry and sport-related lower body injuries. This finding is in line with those of Ruffe et al. (36), who found adolescent male runners with a YBT-UQ SL reach distance of ≥4.0 cm seven times more likely to incur an injury to the lower body. The findings of Ruffe et al. (36) and our findings may be explained by the fact that the YBT-UQ also demands trunk mobility/stability. Limitations in reach directions or asymmetries may lead to decreases in neuromuscular control for the trunk and the lower extremities. Therefore, our results strengthen the notion that injuries are multifactorial (38). Consequently, a multifactorial assessment in the sense of a complex system approach (29) may be better suited as an injury risk screening tool. Overall, the YBT-UQ in isolation may not be sensitive enough to differentiate between low- and high-risk groups to suffer an injury throughout the competitive season. As an additional reason for the lack of relationships between the YBT-UQ results and in-season injury occurrence, the performance of the present sample in terms of YBT-UQ norm values is only slightly above the reported reference values (39). The difference in total performance of the YBT-UQ between those at high risk and those at low risk remained relatively small, making significant results less likely. Further, the YBT-UQ, which assesses upper extremity mobility and stability at the end range of motion, remains a relatively unspecific test concerning the demands placed on the athletes during training and competition. This is especially true as the subjects have full control over their bodies with no perturbations during the YBT-UQ, while during training and games, balance is often challenged by body contact. It is well documented that body contact actions are an essential cause of injuries in handball (9) due to exposure to external forces (5). In addition, based on the notion that injuries are multicausal (40) and due to the possible interaction of many factors (38), weaknesses in one system (e.g., upper extremity mobility and stability as assessed by the YBT-UQ) may be compensated for by other systems.

The match injury rate of 4.5 injuries/1,000 match hours found in the present study is less than those reported by Olsen et al. (3), who found a match injury rate of 8.3 injuries/1,000 match hours in male and 14.5 injuries/1,000 match hours in female U17 players. It is also lower than the results of Møller et al. (4), who found a higher injury incidence in adolescent male and female players, with an incidence of 10.8 in female and 11.5 in male U16 players, as well as 13.0 in female and 17.2 in male U18 players, all per 1,000 match hours. Asai et al. (10) even reported higher incidence rates of 32.7 injuries/1,000 match hours in boys and 20.1 injuries/1,000 match hours in girls (age: 13–14 years). The low incidence of 4.5 injuries per 1,000 match hours in our study may be related to the rather young age (15.7 ± 1.7 years) of this adolescent cohort. A lower injury incidence in this young age group is in line with different studies (3, 4) and a review by Vila et al. (9) that also reported fewer injuries in younger players. Importantly, no conclusions in terms of causation can be drawn from the present results.

### 4.1. Limitations

Different limitations of the study must be addressed. The baseline values of the YBT-UQ were assessed on a single occasion, sometimes referred to as a “static snapshot” (41). However, it is well-reported that upper extremity mobility and stability change over a season (42). Thus, the results of the YBT-UQ at the time of injury might have been different from the preseason values, which we did not control for. In addition, injuries may vary over time during one season (43, 44). Specifically, injury risk differs between the beginning, middle, and end of the season, with the transitions between on- and off-season reported to be risk factors (45), which was partially (see Cox proportional hazard survival regression model) controlled for in the present study. No differentiation in terms of sex and playing position was made to preserve the necessary statistical power of the analyses. However, it is reported that YBT-UQ values significantly differ between playing positions in handball (46, 47). As the mechanisms of injuries were not classified, sub-group analyses on this aspect were not included, which might have given insight in terms of (a) contact vs. non-contact, (b) acute vs. overuse injuries, and (c) the timing of the injuries during the games. The physical parameters of speed, change of direction, agility, balance, strength, and power, all possibly related to injuries, were not assessed. In addition, training quantity and intensity throughout the season were not controlled for, leading to a lack of dose-response assessments (44) for the factor of training load.

## 5. Conclusion

We investigated whether preseason YBT-UQ performance was associated with sport-related injuries during the following competitive season in adolescent handball players. Our results indicate that neither the YBT-UQ reach distances of the throwing or non-throwing arm nor the asymmetry cut-off values (except IL reach asymmetry) were associated with the occurrence of a time-loss injury. Thus, the YBT-UQ seems to have limited benefit as a field-based screening tool for assessment of injury risk in adolescent handball players. Future studies may investigate if the YBT-UQ could be a useful tool when added to a multifactorial test battery. In addition, it would be desirable to include injury mechanisms in terms of contact vs. non-contact, acute vs. chronic, and timing during the games. Moreover, future studies should assess whether the limited association between YBT-UQ results and handball-specific injuries that was demonstrated in our study also applies to other groups of players, i.e., even younger or adult players.

## Data Availability

The raw data supporting the conclusions of this article will be made available by the authors, without undue reservation.
